# Utility of an improved model of amyloid-beta (Aβ_1-42_) toxicity in *Caenorhabditis elegans* for drug screening for Alzheimer’s disease

**DOI:** 10.1186/1750-1326-7-57

**Published:** 2012-11-21

**Authors:** Gawain McColl, Blaine R Roberts, Tara L Pukala, Vijaya B Kenche, Christine M Roberts, Christopher D Link, Timothy M Ryan, Colin L Masters, Kevin J Barnham, Ashley I Bush, Robert A Cherny

**Affiliations:** 1 The Florey Institute of Neuroscience and Mental Health, University of Melbourne, Victoria, 3010, Australia; 2 University of Adelaide, Adelaide, South Australia, Australia; 3 Bio21 Molecular Science and Biotechnology Institute, University of Melbourne, Victoria, Australia; 4 Institute for Behavioral Genetics, University of Colorado, Boulder, CO, USA

**Keywords:** Amyloid beta peptide, Alzheimer’s disease, Caenorhabditis elegans, 8-hydroxyquinoline, PBT2 and drug screen

## Abstract

**Background:**

The definitive indicator of Alzheimer’s disease (AD) pathology is the profuse accumulation of amyloid-ß (Aß) within the brain. Various *in vitro* and cell-based models have been proposed for high throughput drug screening for potential therapeutic benefit in diseases of protein misfolding. *Caenorhabditis elegans* offers a convenient *in vivo* system for examination of Aß accumulation and toxicity in a complex multicellular organism. Ease of culturing and a short life cycle make this animal model well suited to rapid screening of candidate compounds.

**Results:**

We have generated a new transgenic strain of *C. elegans* that expresses full length Aß_1-42_. This strain differs from existing Aß models that predominantly express amino-truncated Aß_3-42_. The Aß_1-42_ is expressed in body wall muscle cells, where it oligomerizes, aggregates and results in severe, and fully penetrant, age progressive-paralysis. The *in vivo* accumulation of Aß_1-42_ also stains positive for amyloid dyes, consistent with *in vivo* fibril formation. The utility of this model for identification of potential protective compounds was examined using the investigational Alzheimer’s therapeutic PBT2, shown to be neuroprotective in mouse models of AD and significantly improve cognition in AD patients. We observed that treatment with PBT2 provided rapid and significant protection against the Aß-induced toxicity in *C. elegans*.

**Conclusion:**

This *C. elegans* model of full length Aß_1-42_ expression can now be adopted for use in screens to rapidly identify and assist in development of potential therapeutics and to study underlying toxic mechanism(s) of Aß.

## Background

One barrier to the efficiency of drug discovery efforts in the area of Alzheimer’s disease (AD) therapeutics is the time and labour intensive nature of animal studies using transgenic mice. Cell based models for high throughput screening of candidate drugs have been proposed to attempt to bridge the gap between cell-free assays and whole animal studies. *Caenorhabditis elegans* offers an efficient *in vivo* system in which to examine the toxic outcomes of over-expression of proteins and peptides that are prone to pathological misfolding [1]. *C. elegans* can be further used as a cost-effective platform for discovering compounds that protect against the toxicity-associated with these misfolded proteins. Simple animal models, like *C. elegans*, do not need to recapitulate all pathological aspects of the respective diseases being modelled to be of use. Indeed, the simplicity of this model may be advantageous; the potentially confounding behavioural and cognitive responses typical of the higher vertebrate are absent. Instead, rapid and clear toxic phenotypes may be preferable for screening strategies, facilitating identification of structure-activity relationships. The well-developed genetics and short life cycle of *C. elegans* allow it to be used in ways that are time and cost-prohibitive in vertebrate systems. As such *C. elegans* represents a complementary tool in drug discovery that may be employed before testing in vertebrate models, to expedite development of new therapeutics.

In order for this model to be useful for drug discovery it must be predictive of efficacy in traditional vertebrate models. In a recent large, unbiased yeast-based screen of over 200,000 compounds in clinical use, the 8-hydroxyquinoline chemical scaffold (8OHQ) was identified as having unique potential to reduce toxicity associated with the aggregation of several neurodegenerative disease-specific proteins [2].

Within the 8OHQs, we have identified PBT2 as a neuro-protective compound that provides rapid cognitive improvement in mouse models of AD [3] and effective in improving cognition and reducing Aß in cerebrospinal fluid in a small Phase IIa trial in AD patients [4]. The exact mode of action of PBT2 is not yet fully defined, however its mechanism is believed to involve a combination of amyloid-beta (Aß) detoxification and metal chaperone activity influencing intracellular homeostasis of biological metals (e.g. Fe, Cu and Zn) [3,5]. Here we describe a *C. elegans* model of AD that would facilitate more rapid testing of compounds to complement the traditional vertebrate (mouse) models for drug discovery.

The key pathological hallmark of AD is the cerebral deposition of plaques composed of Aß peptide [6]. Aß is produced by sequential proteolytic cleavage of the ubiquitously expressed type I transmembrane protein, amyloid ß-protein precursor (APP). Cell and animal based models for AD typically overexpress either APP or its cleavage product Aß. APP is cleaved first by ß-secretase (BACE), and then by γ-secretase, in a heteromeric complex at either plasma or cellular membranes [7]. The Aß released typically ranges from 38 to 43 amino acids in length due to imprecise γ-secretase cleavage, with the predominant species being 40 and 42 amino acids. The accumulation of Aß is thought to lead to disease progression [8], however, the underlying mechanism of Aß toxicity remains unclear.

*C. elegans* express an APP ortholog, APL-1 (Amyloid Precursor-Like-1), but it lacks BACE sites. In addition, the *C. elegans* genome does not appear to encode a BACE ortholog, and to date no Aß-like peptide has been detected in the nematode. *In vivo* effects of transgenic human-Aß can therefore be examined in isolation from APP processing, cleavage or breakdown in this model.

We determined that earlier models of human-Aß expression in *C. elegans* accumulate Aß_3-42_ due to mis-cleavage of a synthetic signal peptide [9]. The truncated Aß_3-42_ has altered *in vitro* biophysical characteristics compared to full length Aß_1-42_, including increased hydrophobicity and propensity to aggregate [9]. However Aß_3-42_ does not significantly contribute to the Aß found in human AD brain. A *C. elegans* model expressing a more disease relevant form of Aß is required in order to more fully exploit this system for drug discovery. Here we describe a new *C. elegans* model that expresses and accumulates full-length Aß_1-42_, and discuss the *in vivo* phenotype. To test the predictive value of this model for identifying protective compounds we then examined the ability of PBT2 to protect against rapid Aß induced toxicity in this animal model.

## Results

### A C. elegans model of Aß_1-42_ expression

**Figure 1:**
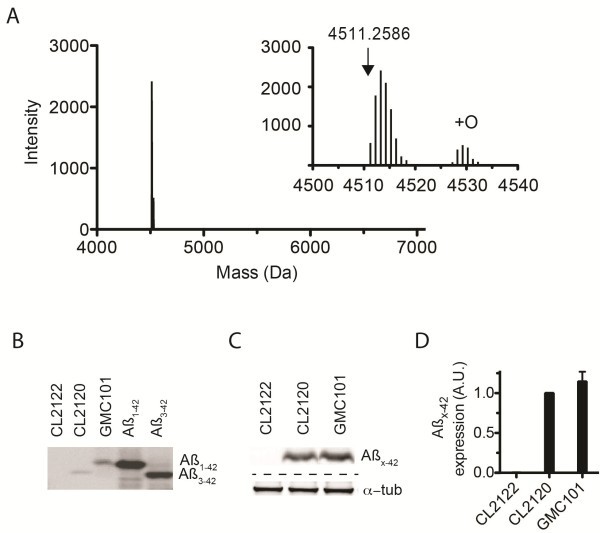
***C. elegans* Aß model accumulates full length Aß**_**1– 42**_**. A** ESI-MS analysis of immuno-precipitated material from *C. elegans* expressing Aß_1-42_. Insert shows an observed species of mono-isotopic molecular weight 4511.2586 (2.5 ppm error) and Aß_1-42_ plus a single oxygen (observed mass 4527.2710). Expected mass of Aß_1-42_ is 4511.2697 Da. **B** TBS soluble lysate from *C. elegans* and synthetic Aβ species resolved on the basis of hydrophobicity and immuno-detected using 4G8 as the primary antibody. Synthetic standards consist of Aβ_1-42_ and Aβ_3-42_. Included are (4-day-old) transgenic control strain CL2122, Aβ_3-42_ expressing CL2120, and Aβ_1-42_ expressing GMC101. **C** Total protein extracts from *C. elegans* resolved via Tricine-SDS-PAGE with immuno-detection of Aß (using 6E10) and α-tubulin. **D** Plot of average total Aß normalized against α-tubulin, shown are means ± SD from *n* = 3 replicates.

To engineer a *C. elegans* strain expressing full length Aß_1-42_ we modified the synthetic signal peptide to be cleaved from the transgenic Aß used in the original expression construct [10]. An extra Asp-Ala (DA) was inserted N-terminal into the human-Aß sequence in the expression vector pCL12(*unc-54*:*Aß*_*1-42*_) [10] (Additional file 1: Figure S1). Predicted processing of the new product, examined (data not shown) via SignalP 3.0 [11], suggested the position of signal peptide cleavage would yield full length Aß_1-42_. Based on this prediction an integrated transgenic strain was engineered in *C. elegans*. Transgene expression was targeted to the bodywall muscle cells, via use of the promoter of *unc-54* (which encodes a heavy chain muscle myosin). We then confirmed the molecular identity of the Aß expressed in the *C. elegans* strain GMC101 via complementary techniques. Using immunocapture and ESI-MS we determined the mono-isotopic molecular weight of the expressed Aß as 4511.2586 Da with an error of 2.5 ppm (Figure 1A). This mass is consistent with full length Aß_1-42_ (expected mass 4511.2697 Da). An additional peak, shifted ~16 Da, was observed corresponding to Aß_1-42_ plus a single oxygen (observed mass 4527.2710 Da). SELDI-TOF-MS incorporating antibody capture also confirmed the expression of Aß_1-42_ (Additional file 2: Figure S2). In addition, bis/Bicine urea PAGE analysis also resolved a single species consistent with Aß_1-42_ (Figure 1B). No additional truncated Aß species were detected. 

The amount of Aß_1-42_ expressed in this new transgenic strain, GMC101, was then compared to that expressing Aß_3-42_ (strain CL2120). Total protein was extracted via a urea-based buffer and the relative amount of Aß was compared via immuno-blot analysis densitrometry (Figure 1C- 1D). An equivalent amount of Aß was detected between strains CL2120 (Aß_3-42_) and GMC101 (Aß_1-42_).

### In situ-aggregation of Aß

**Figure 2:**
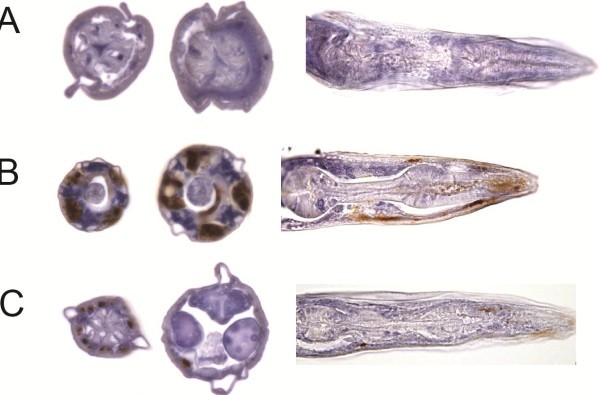
***In vivo* Aß**_**1–42**_** accumulation.** Representative immuno-histochemically stained 5 μm sections (right, middle) of paraffin embedded adult *C. elegans*, showing Aß localization (= brown) in bodywall muscle cells. Shown are transverse (left, middle) and longitudinal sections though the adult head (right) of **A** Transgenic control Strain CL2122, **B** CL2120, expressing Aß_3-42_, and **C** GMC101, expressing Aß_1-42_. Scale bar = 25 μm.

**Figure 3:**
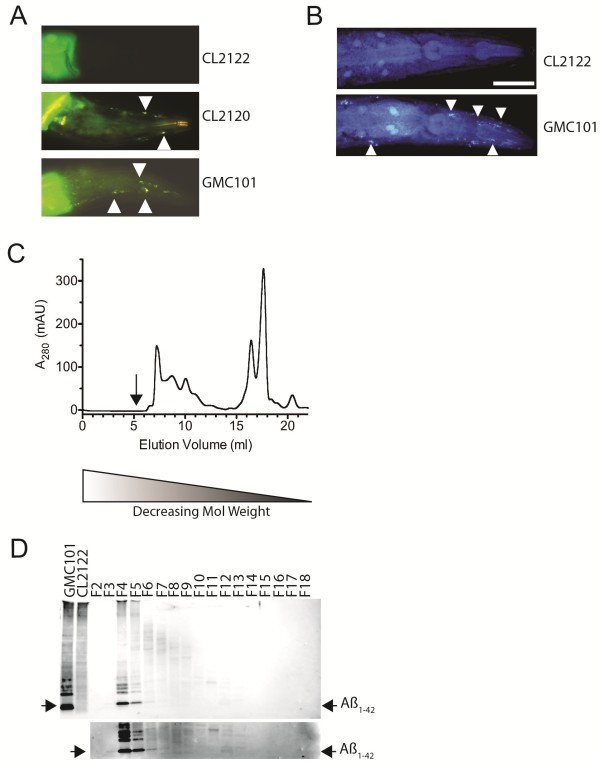
***In vivo* Aß**_**1–42**_** aggregation.** Fluorescence micrographs of adult *C. elegans* heads. **A** ThT positive Aß aggregates are absent in transgenic control strain CL2122 (top), and marked with arrow heads in CL2120, expressing Aß_3-42_ (middle), and GMC101, expressing Aß_1-42_ (bottom). **B** X-34 positive Aß_1-42_ aggregates are seen (arrow head), CL2122 (top) and GMC101 (bottom). Scale bar = 25 μm. **C**. Size-excluded soluble proteins from Aß_1-42_ expressing GMC101. Shown is the absorbance at 280 nm (A_280_) of eluted material against elution volume. Fractions (0.75 ml) were collected from 5.25 ml onwards (marked by arrow). **D** Immuno-blot analysis of size-excluded fractions (F2-18) resolved via 4-12% BisTris SDS PAGE and detected with 6E10. Shown are starting material extracts from strain GMC101 (Aß_1-42_) and control strain CL2122. Aß_1-42_ elutes predominantly in fractions F4-F5. A longer exposure (below) shows Aß also elutes in fractions consistent with lower-order oligomers (i.e. F11-F13).

The cellular localization of the accumulated transgenic Aß_1-42_ was then examined via immuno-histochemistry. Transverse and longitudinal paraffin-embedded 5 μm sections confirmed Aß localization within the bodywall muscle cells (Figure 2), consistent with the promoter activity of the *unc-54* promoter. Fluorescence based immuno-histochemistry also confirmed accumulation of Aß within the bodywall muscle of unsectioned individuals (Additional file 2: Figure S2). The expressed Aß_1-42_ also appears to be at least partially aggregated as deposits stained positive for the amyloidogenic dye Thioflavin T (ThT) (Figure 3A). Aggregated Aß_1-42_ could also be detected *in vivo*, via staining in live animals using the lipophillic-congo red derivative, X-34 [12] (Figure 3B). X-34 has brighter fluorescence than ThT and detects more Aß aggregates than ThT, as has been previously shown [12]. Representative images of the head are shown in Figure 3A- 3B, as this is a region devoid of background fluorescence (including the GFP expressed in the intestine as a co-marker). We noted, however, that dye-binding aggregates are found throughout the entire length of the adult (Additional file 3: Figure S4). 

### Soluble Aß oligomers

Soluble oligomers of Aß have been proposed to be the toxic form of Aß [13]. To explore this further in our *C. elegans* model we used size-exclusion chromatography under native conditions to separate soluble proteins on the basis of size. The expressed Aß_1-42_ in our *C. elegans* model elutes, almost exclusively, as high molecular weight material (>100KDa). Only minor amounts of Aß were detected that eluted in fractions consistent with monomer. These data are consistent with the Aß forming soluble oligomers and/or heteromeric complexes with other cellular macromolecules (e.g. proteins etc).

### Aß_1-42_ toxicity in C. elegans

**Figure 4:**
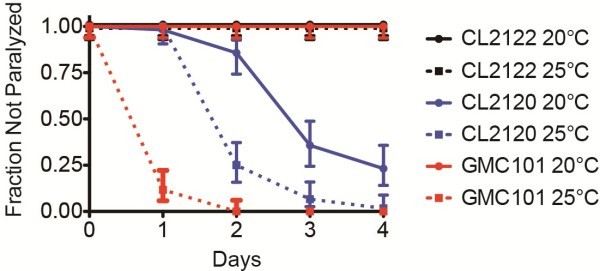
**Aß**_**1–42**_** accumulation causes paralysis.** Age-related paralysis phenotype in Aß_3-42_ and Aß_1-42_ expressing *C. elegans*. Adults shifted to either 20°C or 25°C from the first day of adulthood (day zero) onwards. Plotted are the mean fractions of individuals not paralyzed ± 95% confidence intervals. Shown are control strain CL2122 (20°C *n =* 60; 25°C *n* = 60), Aβ_3-42_ expressing CL2120 (20°C *n* = 56; 25°C *n* = 60), and Aβ_1-42_ expressing GMC101 (20°C *n* = 58; 25°C *n* = 0), where *n* = number of individuals assayed. Plot shown is representative of 4 experiments.

Differences in transgene array copy number and the genomic insertion sites of the trangenic arrays limits the ability to directly compare phenotypes between strains. However, we observed similar yet distinct toxic effects from Aß_1-42_ versus Aß_3-42_. The expression of Aß_1-42_ had no adverse effects on motility if adults were cultured and maintained at 20°C over 4 days post adulthood (Figure 4B). In contrast, *C. elegans* expressing Aß_3-42_ show an age-dependent paralysis phenotype when cultured at 20°C. However, when Aß_1-42_ expressing adults, developed at 20°C, were shifted to 25°C we observed severe and fully penetrant paralysis within 48 h. The paralysis at 25°C appeared more severe for those expressing Aß_1-42_ compared to Aß_3-42_.

### Protection against Aß-induced paralysis by an investigational drug

**Figure 5:**
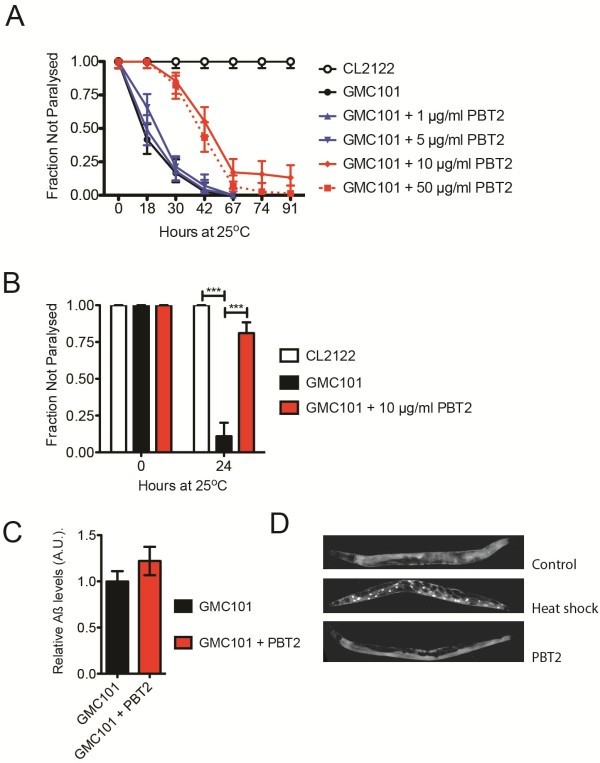
**PBT2 protects against Aß**_**1–42**_** induced toxicity.** Paralysis associated with Aß_1-42_ expression is protected by PBT2. Plotted are the proportions of individuals not paralysed with upper and lower 95% confidence intervals. **A** PBT2 at 10 μg/ml was found to be most effective when cultures were exposed to compound for 24 prior to the initiation of the assay. **B** PBT2 effects are very rapid, such that immediate exposure to 10 μg/ml PBT2 suppressed Aß-induced paralysis. Control strain CL2122 (*n* = 74), Aß_1-42_ strain GMC101 (*n* = 73), GMC101 strain + PBT2 (*n* = 74), where *n* = number of individuals assayed and *** *p* < 0.001. Plot shown is representative of 3 experiments. **C** Exposure to 10 μg/ml PBT2 for 24 h did not significantly alter total Aß levels. Shown is mean ± SD quantitation via densitrometry of *n* = 3 immuno-blot analyses. **D** Exposure to PBT2 does not alter DAF-16 localization. Shown are representative epifluorescence micrographs of DAF-16: GFP cytoplasmic localization in Control and PBT2 (10 μg/ml for 24 h) treated populations in contrast to nuclear localization in samples heat-shocked for 2 h at 35°C.

The rapid paralysis from Aß_1-42_ expression in this *C. elegans* strain is well suited for assessing drug effects. To explore the utility of this nematode model of Aß toxicity to identify protective compounds we examined the effect of PBT2. We exposed L4 (the final larval stage) to a range of PBT2 concentrations for 24 h prior to a shift to 25°C (Figure 5A). A dose of 10 μg/ml was found to offer significant protection against the Aß-induced paralysis. A protective effect of PBT2 was observed after one day at 25°C with significantly fewer individuals exhibiting paralysis (*p* < 0.001). To examine how quickly PBT2 could act we then minimized the exposure time of cultures prior to the temperature shift to 25°C. When young adults (3 days post egg lay) were exposed to PBT2 and at the same time were shifted to 25°C, significant protection was still observed (*p* < 0.001, Figure 5B). PBT2 effects on Aß toxicity therefore appear to be rapid and are unlikely to be due to indirect effects on development. Treatment for with PBT2 for 24 h did not affect total Aß levels (Figure 5C), or *in vivo* aggregated Aß as determined by X-34 staining (data not shown).

Reduction of insulin-like signalling (ILS) in *C. elegans* is known to protect against Aß toxicity [14]. To explore whether PBT2 acts via modulation of the ILS pathway in *C. elegans* we used a GFP-based reporter strain. Under conditions of lowered ILS or stress (e.g. starvation, heat shock, etc) DAF-16 (a FOXO transcription factor) translocates from the cytoplasm to the nucleus [15]. Exposure to PBT2 did not alter DAF-16 localization (Figure 5D), suggesting its effects are not via modulation of ILS. We also observed that exposure to PBT2 produced no detectable induction of small heat shock proteins (data not shown), as measured by a *hsp-16.2*:GFP reporter strain [16]. This data are consistent with the protective effects of PBT2 being derived by a mechanism other than a generalized stress response.

## Discussion

Previously, we demonstrated that the existing Aß models in *C. elegans* accumulate amino truncated Aß_3-42_ instead of Aß_1-42_[9]. The Aß_3-42_ peptide has different physicochemical properties to Aß_1-42_; Aß_3-42_ is more hydrophobic and aggregates more rapidly *in vitro*. In human AD brain Aß_1-42_ is a predominant Aß species [17], along with additional various N- and C-terminal variants [6,18,19]. We generated a new transgenic model of *C. elegans* that accumulates full-length hu-Aß_1-42_ peptide. To achieve correct signal peptide cleavage from the Aß_1-42_ we inserted two additional amino acids (-DA-) between the synthetic signal peptide and the Aß peptide corrected the cleavage, resulting in expression of full length Aß_1-42_. A similar approach was used to correct signal peptide cleavage from Aß_1-42_ in an expression construct stably transfected into COS7 cells line [20].

In our model the Aß_1-42_ is expressed in body wall muscle cells, where it aggregates and results in severe and fully penetrant age progressive-paralysis (Figure 4) at 25°C. Anecdotal observations suggest that paralysis from Aß_1-42_ is more rapid than that caused from Aß_3-42_ expression. Previous studies have reported a related phenotype of reduced motility in liquid for the Aß_3-42_ expressing strain [21].

Disease severity of AD correlates with soluble (i.e. soluble in an aqueous buffer such as phosphate- or tris-buffered saline) but not aggregated (plaque)-Aß [22]. Several studies suggest that soluble oligomers are likely to be the toxic form of Aß [13]. However, the extent to which soluble Aß-oligomers exist *in vivo* is not clear. Observation of Aß-oligomers in extracts resolved via sodium dodecyl sulfate-polyacrylamide gel electrophoresis (SDS-PAGE) has questionable relevance due to Aß self-interaction induced by SDS [23,24]. As an alternative approach we have used liquid chromatography under native conditions to size exclude proteins and have observed that Aß_1-42_ in *C. elegans* elutes as high-molecular-weight species (>100 KDa) consistent with high order oligomers. Previous studies using Aß_3-42_ expressing *C. elegans* also suggest soluble Aß-oligomers form and correlate with toxicity, rather than aggregated Aß [14]. The precise molecular identity of the toxic Aß species in AD brain or animal models of Aß toxicity, and their cellular target(s) are yet to be established [25].

Treatment with ThT of *C. elegans* expressing Aß_3-42_ suppresses Aß-toxicty [26]. As ThT binds fibirils this suggests that aggregation may influence Aß toxicity. However, the ThT effects on additional stress phenotypes are dependent on HSF-1 and SKN-1, both of which are stress response transcription factors. This suggests that ThT may lessen Aß-toxicity indirectly via off-target stress response pathways. Expression of Aß in *C. elegans* increases oxidative stress, which occurs prior to detection of Aß fibril formation [27]. This is consistent with the idea that the molecular species responsible for Aß toxicity is pre-fibrillar. Furthermore, single amino acid substitutions (e.g. Leu17Pro and Met35Cys) blocked fibril formation in *C. elegans* but do not reduce toxicity [28], suggesting that fibrillar-Aß itself is not the toxic species.

This whole-animal model of Aß_1-42_ toxicity is well suited to studies of drug intervention. Assays of paralysis are rapid (approximately 4 days in total), with a clear and robust phenotype. PBT2, a drug undergoing clinical investigation for AD, was found to protect *C. elegans* against Aß_1-42_ toxicity. This effect is consistent with neuro-protection reported in AD patients [4] and mouse AD models [3] and supports the utility of this nematode model for drug discovery. This model can also provide useful information on mechanism of action of candidate drugs. For example, previous cell culture experiments reported that PBT2 lowered total Aß via up-regulated matrix metalloproteases [29]. In contrast, we observed that Aß levels were not affected despite suppression of Aß-toxicity, suggesting that this animal model has identified additional modes of drug action.

## Conclusions

This new *C. elegans* model of Aß_1-42_ toxicity has utility for screening of compound libraries, serving as a bridge between high throughput *in vitro* assays and time and labour intensive transgenic mouse trials. The turnaround time of 4 days compared with weeks to months for the typical mouse study (excluding the months of maintenance and husbandry while the animals reach the appropriate age) favours this model as a cost effective method of identifying lead compounds for more intensive investigation. This model also provides a useful tool to explore the mechanism(s) of Aß_1-42_ toxicity.

## Methods

### Strains

The strains N2, wild type; CL2120, *dvIs14*(pCL12(*unc-54*: *hu-Aß*_*1–42*_) + pCL26(*mtl-2*: GFP)), CL2122; *dvIs15*(*mtl-2*: GFP) [28], CL2070, *dvIs70*(pCL25(*hsp16.*2:: GFP) + pRF4(*rol-6*(*su1006*)) [16] and TJ356; *zIs356*(*daf-16*:: DAF-16-GFP) + pRF4(*rol-6*(*su1006*)) [15] were obtained from the *Caenorhabditis* Genetics Center. To engineer a full-length *Aß*_*1–42*_ expressing strain the pCL12 plasmid [10] was modified by the addition of codons (5’-GAC-CGC-3’) for residues ASP-ALA between the signal peptide and the *Aß*_*1–42*_ ORF via a QuikChange Multi Site-Directed Mutagenesis Kit (Stratagene). The primers used were: 5'-gcaccagcaggtaccgacgcggatgcagaattccga, and 5'-tcggaattctgcatccgcgtcggtacctgctggtgc. The resulting plasmid, called pCL354 (*unc-54*:*DA-Aß*_*1-42*_) shown in Additional file 1: Figure S1, encodes: MHKVLLALFFIFLAPAGTDA*DAEFRHDSGYEVHHQKLVFFAEDVGSNKGAIIGLMVGGVVIA,* where the inserted residues are underlined and Aß_1-42_ sequence shown in italics. A transgenic strain was generated via gonad micro-injection and a stable integrant derived following γ-irradiation as previously described [28]. This strain was then back crossed to wild type four times to give GMC101, *dvIs100* [pCL354(*unc-54*:*DA-Aß*_*1-42*_) + pCL26(*mtl-2*: *GFP*)]. All strains were cultured at 20°C on NGM [30] or 8P media [31] with *E. coli* (strain OP50) as indicated. On the first day of adulthood (3-days-old), populations were aged at 20 or 25°C as indicated.

### Immunoprecipitation

To a ~100 mg liquid-N2 frozen pellet of 5-day-old GMC101 adults 2:1 (v/w) of 70% formic acid was added and incubated for 4 hrs at room temperature. The lysate was centrifuged at 16,500 × *g* for 15 min, the supernatant retained and neutralized with 1:20 (v/v) 1 M Tris pH 8.0 and then diluted again 1:10 (v/v) in H_2_0. For immunocapture W0-2 (epitope: Aß5-8) [32] antibody (50 μg) was bound to 2 mg of dynabeads as per manufacturers instructions (Invitrogen). Subsequent immunocapture, washing and elution steps were performed following the manufacturer's instructions. The eluted material was vacuum-centrifuged to dryness then resuspended in 10 μL of 6 M urea and 5% acetic acid at room temperature for 10 min, and then desalted and concentrated through a C_4_ ZipTip (Millipore) for mass spectrometry.

### Mass spectrometry

MS measurements were performed on a LTQ-Orbitrap (Thermo Scientific) operated in the positive ion mode, with the sample introduced by nano-electrospray from borosilicate capillaries (New Objective). Typical instrumental parameters included; ionisiation spray voltage, 1.5 kV; capillary voltage, 40 V; tube lens voltage, 60 V; capillary temperature; 300°C; maximum injection time, 100 ms; orbitrap mass resolution, 100000 (at m/z 400); acquisition time, 1-2 min. Spectra were deconvoluted and analysed using Qual Browser v.2.0 software (Thermo Scientific).

SELDI-TOF-MS analysis was also performed on TBS soluble material as previously described [9]. Immunocapture was performed using affinity-purified W0-2 (epitope: Aß5-8) [32] antibody coupled to ProteinChip PS10 arrays (Bio-Rad).

### Immunoblot analysis

For separation of Aß based on peptide hydrophobicity [33] bis/bicine urea-PAGE analysis was performed as previously described [9] but modified for a 20 × 20 cm Protean II xi system (BioRad). Affinity purified 4G8 (epitope: Aß18–22, Signet Laboratories) primary antibody was used at 1 μg/ml.

For comparison of Aß levels ~1000 adults were collected in S-basal [34] in triplicate, then frozen in liquid-N_2_. Samples were then extracted in 3 volumes of urea buffer (7 M urea, 2 M Thiourea, 4% w/v CHAPS, 1.5% w/v dithiothreitol and 50 mM Tris pH 8.0) disrupted via sonication, and then centrifuged at 16,500 × *g* for 10 min. A 10 μl sample of the supernatant was added to 3 μl of loading buffer (10% v/v glycerol, 250 mM Tris pH 8.5, 2% w/v SDS, 0.5 mM EDTA and 0.2 mM Orange-G) and reduced with 1/10 volumes of 0.5 M dithiothreitol. Samples were then heated at 70°C for 10 min, mixed and centrifuged at 13,000 g for 1 min. Material was resolved via Tricine-SDS-PAGE (16%/6M Urea) [35] then transferred to nitrocellulose membranes, boiled for 3 min (via a microwave oven) in PBS pH 7.4 and blocked for 1 h at room temperature in 0.5% (w/v) skim milk. Membranes were probed overnight at 4°C with or 6E10 (epitope: Aß4–9, Sigma) at 1 μg/ml as indicated. Blots were re-probed with anti-α-tubulin (Sigma T6074, 1:10000) to standardize total protein loading. Standard enhanced chemiluminescence was then performed [9].

### Immunohistochemistry

*C. elegans* were washed in S-basal [34], fixed overnight in 10% (v/v) Neutral Buffered Formalin (NBF) at 4°C, embedded in agar (2% w/v in phosphate buffered saline) blocks and then fixed again in 10% NBF overnight. Following processing of the agar blocks into paraffin, 5 μm sections were prepared, deparaffinised and treated with 90% formic acid (FA) prior to Aß immunohistochemistry with a 1:200 dilution of 1E8 mouse monoclonal (SmithKline Beecham) antibody (epitope: Aß18–22). Antibody binding sites were detected with a peroxidase labelled streptavidin biotin system (Dako K0675) with a 3,3’-diaminobenzidine tetrahydrochloride (DAB) chromogen (Dako) resulting in a brown reaction product. Samples were counter stained with Harris Haematoxylin solution (Amber Scientific).

### Size exclusion chromatography

To a frozen pellet of *C. elegans* 1:1 (w/v) volumes of PBS (pH7.4) with proteinase inhibitors added (Roche Applied Science) was added, then disrupted by sonnication using 6 cycles of 6 sec ‘on’, 10 sec ‘off’ with a 40% duty cycle. Following centrifugation at 100000 × g for 30 min at 4°C the soluble fraction was collected and then diluted to 10mg/ml. A single 100 μl injection of 1 mg total protein into a Superdex 75 10/300 GL gel filtration column was size excluded in PBS pH7.4 at a flow rate of 0.75 ml/min using an Agilent 1200 HPLC. Fractions of 1 ml were collected and subsequently analysed by 4-12% BisTris SDS-PAGE and immunoblot as above using affinity purified 6E10 antibody as above.

### *Synthesis of X-34* (1,4-bis(3-carboxy-4-hydroxyphenylethenyl)-benzene)

A modified procedure of Styren *et al*[12] was used, where a mixture of 5-formylsalicylic acid (2.2 mmol), *p*-xylylenediphosphonic acid tetraethylester (1 mmol) and potassium *tert*-butoxide (10 mmol) in anhydrous dimethylformamide (10 mL) was stirred at 40°C for 16 h. The reaction mixture was cooled to room temperature and poured into ice-water to give yellow precipitate which was isolated by filtration. The yellow solid was further washed with diethyl ether and dried to give 265 mg of the required product. ^1^H NMR (500 MHz , d_6_-DMSO): d 7.97 (d, J = 2 Hz, 2H), 7.79 (dd, J = 8.5, 2 Hz, 2H), 7.56 (s, 4H), 7.25 (d, J = 16 Hz, 2H), 7.11 (d, J = 16 Hz, 2H), 6.96 (d, J = 8.5 Hz, 2H). MS/EI: 403 (M + 1).

The differential changes in X-34 fluorescence between freshly refolded and fibrillar Aß_1-42_ was confirmed by obtaining emission (excitation: 350 nm) and excitation (emission: 490 nm) spectra with a Flexstation 3 plate reader (Molecular Devices) equipped with monochromators, using an average of 15 reads and an integration time of 2 seconds (Additional file 4: Figure S3).

### Microscopy

Thioflavin T (ThT) staining of 10% Neutral Buffer Formalin-fixed samples [28] and X-34 *in vivo* staining in live *C. elegans* samples [36] were performed as previously described, using a Leica DM2500. Immunohistochemistry on whole *C. elegans* was performed using 6E10 (epitope: Aß4–9) antibody and counter staining of nuclei using 4',6-diamidino-2-phenylindole (DAPI) was performed using standard protocols [10].

### Paralysis assay

All populations were cultured at 20°C and developmentally synchronized from a 4 h egg-lay. At 64-72 h post egg-lay (time zero) individuals were shifted to 20°C or 25°C, and body movement assessed over time as indicated. Nematodes were scored as paralysed if they failed to complete full body movement (i.e a point of inflection traversing the entire body length) either spontaneously or touch-provoked. Proportion of individuals not paralysed were calculated and confidence intervals determined without a correction for continuity [37]. Comparisons of proportions were made using a 2-tailed Z-test. Experiments were replicated as indicated.

### Compound effects

PBT-2 (Prana Biotechnology, Australia) was dissolved in ethanol (<1 ml) and added to molten NGM (at 55°C) at the concentrations described, no compound controls included a corresponding volume of ethanol. In addition all media (control and compound) contained 50 μg/ml Ampicillin (Sigma) to suppress bacteriological activity. All media was stored at 4°C and used within one-week. *C. elegans* cultures were transferred onto media with compound as L4 larvae (48 h post egg lay) for 24 h at 20°C. Cultures were then transferred to 25°C as young adults (time zero) and scored for paralysis as described above.

## Abbreviations

Aß: amyloid beta peptide; AD: Alzheimer’s disease; 8OHQ: 8-Hydroxy quinoline; ESI-MS: electrospray ionization-mass spectrometry; ThT: Thioflavin-T; X-34: 1,4-bis(3-carboxy-4-hydroxyphenylethenyl)-benzene; SDS-PAGE: sodium dodecyl sulfate-polyacrylamide gel electrophoresis.

## Competing interests

CLM, KJB and RAC are consultants for Prana Biotechnology Ltd.

## Authors’ contributions

GM conceived and designed the research; GM, BRR, TLP, TMR, CMR, CDL and VBK performed research; GM, TLP and CDL analysed data; CLM, KJB, AIB and RAC provided material support; GM wrote the paper. All authors read and approved the final manuscript.

## Supplementary Material

Additional file 1 **Figure S1.** A DNA sequence (4982 bp) of plasmid pCL354(*unc-54:DA-Aß1-42*). Shown is the DA-insert codons in red and Aß1-42 ORF in green. Click here for file

Additional file 2 **Figure S2.** A SELDI-TOF-MS analysis of TBS lysate from *C. elegans* expressing Aß1-42. A peptide species with an average m/z of 4511.6 Da (775 ppm error) corresponds to Aß1-42 (calculated average M + H + 4515.1). This estimate is within the typical error associated with SELDI-TOF-MS. B Epifluorescence micrograph of adult GMC101 head showing immunolocalization of Aß1-42 (red) with DAPI stained nuclei (blue). Click here for file

Additional file 3 **Figure S4.** Epi-fluorescence micrograph of *C. elegans* expressing Aß1-42. A. Live imaging of aggregated Aß using X-34. Dye-binding aggregates (arrowheads) can be seen throughout the entire body length. Scale bar = 25μm. B. ThT also binds Aß aggregates (arrowheads) throughout adult *C. elegans*. Click here for file

Additional file 4 **Figure S3.** Fluorescence properties of X-34. Excitation (emission wavelength of 490 nm) and emission spectra (using excitation wavelength of 350nm) were acquired for X-34 in the presence of freshly refolded (black) and fibrillar Aß1-42 (blue) with a step size of 2 nm. This analysis indicates a greater than 1000 fold increase in fluorescence intensity in the presence of fibrillar Aß1-42. Click here for file
