# Supplementary material for: Utility of an improved model of amyloid-beta (Aβ1-42) toxicity in Caenorhabditis elegans for drug screening for Alzheimer’s disease
Source: Mol Neurodegener. 2012 Nov 21;7:57. doi: 10.1186/1750-1326-7-57 (PMC3519830; doi:10.1186/1750-1326-7-57)
Supplement: Additional file 1 — Figure S1. A DNA sequence (4982 bp) of plasmid pCL354(unc-54:DA-Aß1-42). Shown is the DA-insert codons in red and Aß1-42 ORF in green. [file 1750-1326-7-57-S1.pdf]

pCL354 sequence

ATGACCATGATTACGCCAAGCTTGTCTTCTTCTAAATTCCCATAAAATCCCGAAACTCCTTCCCTCTATCTTCTTTTTCTTCTCGTTTTCAAATGTTTCTCTCTATCCCATTCTCTCATC
AATTGAGTGGGATGAGGCTATCTCTGCCTCTCTTCTGAATCTCTGAACCATCTTACATTACACTGTGGATGACGAGCCCCACAGGCTCCCTTGCATCAGATACTGCCATTGGGGATGGCA
AAGAAGAGAGAAGGTATTGTGAGGATATATTTTTCTAAGAAAAAACGTTTGAAGAAAAGAAGATGAAGAAGATCTGCTTGATTCATTGCACAAGTTAGAAGTAACAGGGGTCTATATTTC
GAAGAACTTAAAGGGAATGCAACTGAACATAAAATTAAACAAAGGGATTGAATCCTGCAGTGAGTATTTTCGGTTTTTCACTGGTTCTCTGTAAAAAGAGTAATGCAAAGGGCAAGTTAA
CTTAGGTCGTAAATGTATTGAATTTGCTTAAAATCTGAAGATCTAGTGGTGAACCGTGGAAGATTATCAAGAGGAGGCTGAAGATCTGTTTAAGAACCATTAATCAAACTGGTATTCTAT
TTTCACTGGTTGTATGTAAACATTCTATCTTATTCCTTTTATCACTGTTCTGCACTTTCCTATAAAAAAAGTTGACCGACCGTACTCTCTGAATTCATTTTTCCCGATCTTACCAACTCC
CGATCTATCTCTATCCCTGGTTTTTTCTTCGTGCTCCAATGGAATTCTTGAGACTTCCACTATCTTCTCTGGCACCCTCCACTACGCGTAGGCGTCTCTCGCTTCGTGTATTCCCGGGAA
GCCGGTTCCCGTCTCTCCCGCCGCTGCCGCTGCCGCACACAGCTTTACACCTCGTAGAATCCCCAAAGAGGGGCGTGGCTTGCGGGTGCCAACATCCTCCTGCCGAGGAAGAAGCAGGCA
CTCATCACTCGCATCATCAACCTCGGGATTGGCCAAAGGACCCAAAGgtatgtttcgaatgatactaacataacatagaacattttcagGAGGACCCTTGgctagcaaaaatgcataagg
ttttgctggcactgttctttatctttctggcaccagcaggtacc**GACGCG**GATGCAGAATTCCGACATGACTCAGGATATGAAGTTCATCATCAAAAATTGGTGTTCTTTGCAGAAGATG
TGGGTTCAAACAAAGGTGCAATCATTGGACTCATGGTGGGCGGTGTTGTCATAGCGTGAgctccgcatcggccgctgtcatcagatcgccatctcgcgcccgtgcctctgacttctaagt
ccaattactcttcaacatccctacatgctctttctccctgtgctcccacccccctattttttgttattatcaaaaaaacttcttcttaatttctttgtttttttagcttcttttaagtcacct
ctaacaatgaaattgtgtagattcaaaaatagaattaattcgtaataaaaagtcgaaaaaaaattgtgctccctcccccccattaataataattctatcccaaaatctacacaatgttctgt
gtacacttcttatgttttttttacttctgataaatttttttttgaaacatcatagaaaaaaccgcacacaaaataccttatcatatgttacgtttcagtttatgaccgcaatttttatttc
ttcgcacgtctgggcctctcatgacgtcaaatcatgctcatcgtgaaaaagttttggagtatttttggaatttttcaatcaagtgaaagtttatgaaattaatttcctgcttttgcttt
ttgggggtttcccctattgtttgtcaagagtttcgaggacggcgtttttcttgctaaaatcacaagtattgatgagcacgatgcaagaaagatcggaagaaggtttgggtttgaggctca
gtggaaggtgagtagaagttgataatttgaaagtggagtagtgtctatggggtttttgccttaaatgacagaatacattcccaatataccaaacataactgtttaaaattaaacattttt
ctaaattttatatgatttcttttaaatttgcaaaaattacttaaatttgaattcccgcgcaaatgagtgacttcattttctgcattattgtgttttccggctatattaataggtatttgt
ttgtgtttttctttattttatgattcgaactccaatttgtaaattttcgaacatatttccctaaagaaaaaatatgattaatctggaaaaattggaaaattatttttcaaataaaaaaca
aagaaaaaaatgaagaaaaacctattagtttggccataaaacgcaaaaatgtcgaaaatgacgtcactcatctgcgcgggaaatcaagaataattcggccttttttatttttttggaaaa
tcgtaaaacatttagaaaaatttttaatagttatagtgggactgtattctgtcatttagggcaaaagccagagacgctactccaccgttgggggatccactagtcggccgtacgggccc
tttcgtctcgcgcgtttcggtgatgacggtgaaaacctctgacacatgcagctcccggagacggtcacagcttgtctgtaagcggatgccgggagcagacaagcccgtcagggcgcgtca
gcgggtgttggcgggtgtcggggctggcttaactatgcggcatcagagcagattgtactgagagtgcaccatatgcggtgtgaaataccgcacagatgcgtaaggagaaaataccgcatc
aggcggccttaagggcctcgtgatacgcctatttttataggttaatgtcatgataataatggtttcttagacgtcaggtggcacttttcggggaaatgtgcgcggaacccctatttgttt
atttttctaaatacattcaaatatgtatccgctcatgagacaataaccctgataaatgcttcaataatattgaaaaaggaagagtatgagtattcaacatttccgtgtcgcccttattcc
cttttttgcggcattttgccttcctgtttttgctcacccagaaacgctggtgaaagtaaaagatgctgaagatcagttgggtgcacgagtgggttacatcgaactggatctcaacagcgg
taagatccttgagagttttcgccccgaagaacgttttccaatgatgagcacttttaaagttctgctatgtggcgcggtattatcccgtattgacgccgggcaagagcaactcggtcgccg
catacactattctcagaatgacttggttgagtactcaccagtcacagaaaagcatcttacggatggcatgacagtaagagaattatgcagtgctgccataaccatgagtgataacactgc
ggccaacttacttctgacaacgatcggaggaccgaaggagctaaccgcttttttgcacaacatgggggatcatgtaactcgccttgatcgttgggaaccggagctgaatgaagccatacc
aaacgacgagcgtgacaccacgatgcctgtagcaatggcaacaacgttgcgcaaactattaactggcgaactacttactctagcttcccggcaacaattaatagactggatggaggcgga
taaagttgcaggaccacttctgcgctcggcccttccggctggctggtttattgctgataaatctggagccggtgagcgtgggtctcgcggtatcattgcagcactggggccagatggtaa
gccctcccgtatcgtagttatctacacgacggggagtcaggcaactatggatgaacgaaatagacagatcgctgagataggtgcctcactgattaagcattggtaactgtcagaccaagt
ttactcatatatactttagattgatttaaaacttcatttttaatttaaaaggatctaggtgaagatcctttttgataatctcatgaccaaaatcccttaacgtgagttttcgttccactg
agcgtcagaccccgtagaaaagatcaaaggatcttcttgagatcctttttttctgcgcgtaatctgctgcttgcaaacaaaaaaaccaccgctaccagcggtggtttgtttgccggatca
agagctaccaactctttttccgaaggtaactggcttcagcagagcgcagataccaaatactgtccttctagtgtagccgtagttaggccaccacttcaagaactctgtagcaccgcctac
atacctcgctctgctaatcctgttaccagtggctgctgccagtggcgataagtcgtgtcttaccgggttggactcaagacgatagttaccggataaggcgcagcggtcgggctgaacggg
gggttcgtgcacacagcccagcttggagcgaacgacctacaccgaactgagatacctacagcgtgagcattgagaaagcgccacgcttcccgaagggagaaaggcggacaggtatccggt
aagcggcagggtcggaacaggagagcgcacgagggagcttccagggggaaacgcctggtatctttatagtcctgtcgggtttcgccacctctgacttgagcgtcgatttttgtgatgctc
gtcaggggggcggagcctatggaaaaacgccagcaacgcggcctttttacggttcctggccttttgctggccttttgctcacatgttctttcctgcgttatcccctgattctgtggataa
ccgtattaccgcctttgagtgagctgataccgctcgccgcagccgaacgaccgagcgcagcgagtcagtgagcgaggaagcggaagagcgcccaatacgcaaaccgcctctccccgcgcg
ttggccgattcattaatgcagctggcacgacaggtttcccgactggaaagcgggcagtgagcgcaacgcaattaatgtgagttagctcactcattaggcaccccaggctttacactttat
gcttccggctcgtatgttgtgtggaattgtgagcggataacaatttcacacaggaaacagct

Fig S1
