# Supplementary figures and images for: Utility of an improved model of amyloid-beta (Aβ1-42) toxicity in Caenorhabditis elegans for drug screening for Alzheimer’s disease
Source: Mol Neurodegener. 2012 Nov 21;7:57. doi: 10.1186/1750-1326-7-57 (PMC3519830; doi:10.1186/1750-1326-7-57)

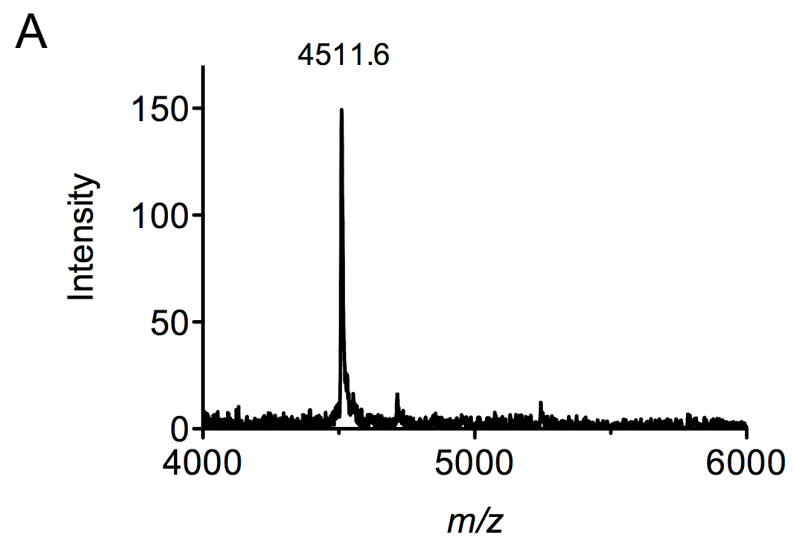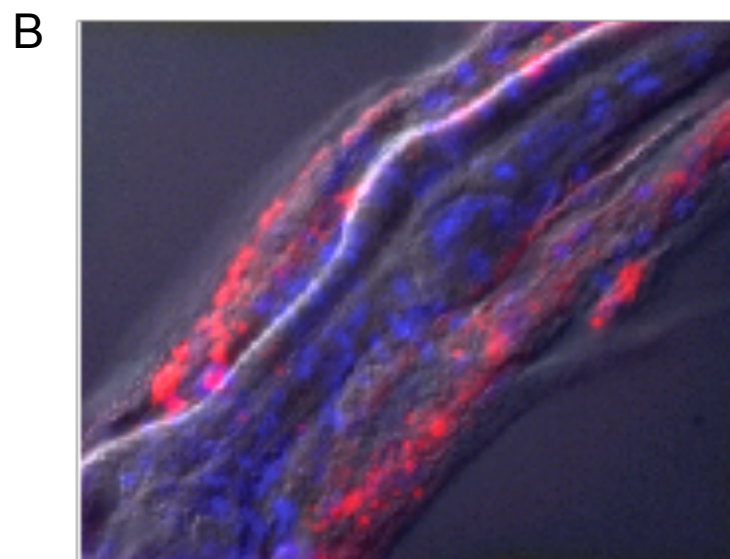

Fig S2

Supplement: Additional file 2 — Figure S2. A SELDI-TOF-MS analysis of TBS lysate from C. elegans expressing Aß1-42. A peptide species with an average m/z of 4511.6 Da (775 ppm error) corresponds to Aß1-42 (calculated average M + H + 4515.1). This estimate is within the typical error associated with SELDI-TOF-MS. B Epifluorescence micrograph of adult GMC101 head showing immunolocalization of Aß1-42 (red) with DAPI stained nuclei (blue). [file 1750-1326-7-57-S2.pdf]

A

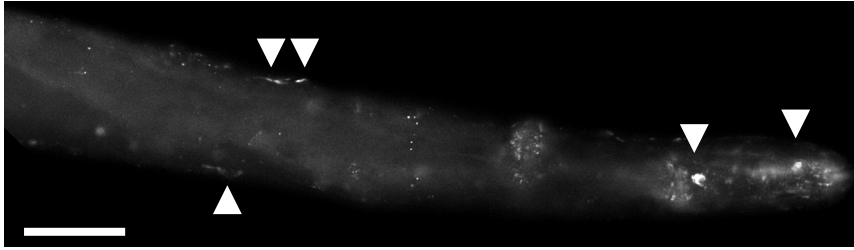

B

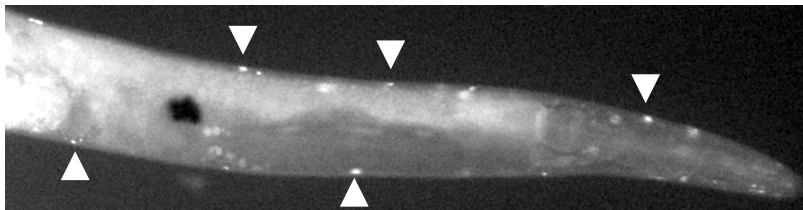

Supplement: Additional file 3 — Figure S4. Epi-fluorescence micrograph of C. elegans expressing Aß1-42. A. Live imaging of aggregated Aß using X-34. Dye-binding aggregates (arrowheads) can be seen throughout the entire body length. Scale bar = 25μm. B. ThT also binds Aß aggregates (arrowheads) throughout adult C. elegans. [file 1750-1326-7-57-S3.pdf]

A

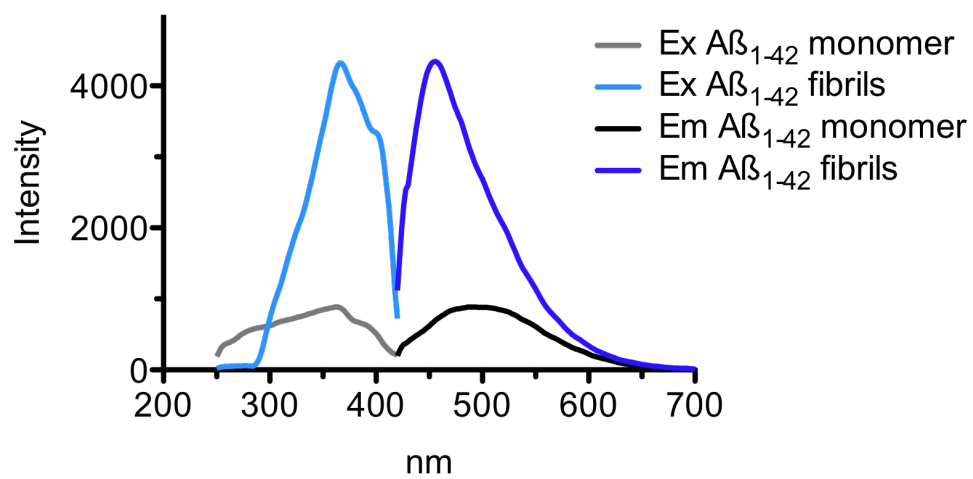

Fig S3

Supplement: Additional file 4 — Figure S3. Fluorescence properties of X-34. Excitation (emission wavelength of 490 nm) and emission spectra (using excitation wavelength of 350nm) were acquired for X-34 in the presence of freshly refolded (black) and fibrillar Aß1-42 (blue) with a step size of 2 nm. This analysis indicates a greater than 1000 fold increase in fluorescence intensity in the presence of fibrillar Aß1-42. [file 1750-1326-7-57-S4.pdf]
